# Automatic differentiation of voluntary and tremulous motion using ensemble empirical mode decomposition and convolutional Bi-directional LSTM

**DOI:** 10.1038/s41598-025-08216-7

**Published:** 2025-10-08

**Authors:** Jie Qi Ling, A. S. M. Bakibillah, Ping Yi Chan, Surya G. Nurzaman, Chee Pin Tan

**Affiliations:** 1 School of Engineering, Monash University Malaysia, Jalan Lagoon Selatan, Bandar Sunway, 47500 Selangor Malaysia; 2 Department of Systems and Control Engineering, School of Engineering, Institute of Science Tokyo, Tokyo, 152-8552 Japan; 3 Medical Engineering & Technology Hub, School of Engineering, Monash University Malaysia, Jalan Lagoon Selatan, Bandar Sunway, 47500 Selangor Malaysia; 4 Centre for Net-Zero Technology, Monash University Malaysia, Jalan Lagoon Selatan, Bandar Sunway, 47500 Selangor Malaysia

**Keywords:** Parkinson’s disease, Parkinsonian tremor, Hand-arm orientation data, Ensemble empirical mode decomposition, Convolutional bi-directional LSTM, Automatic differentiation, Neurological disorders, Parkinson's disease, Biomedical engineering, Data processing, Machine learning

## Abstract

To develop applications for assisting Parkinson’s disease (PD) patients, extracting Parkinsonian tremors from the raw signal is crucial; however, conventional methods such as filtering require a preset frequency range, and a poorly set frequency range may lead to the inclusion of undesired signals. AI algorithms can potentially overcome the heterogenous tremor characteristics in different patients, but they have been applied in the disease or tremor type classification rather than in tremulous-voluntary motion classification. Hence, this study presents an approach to automatically differentiate between voluntary and tremulous motions in PD patients, achieved by combining ensemble empirical mode decomposition (EEMD) and convolutional bi-directional long short-term memory (LSTM). Non-labelled raw hand-arm orientation data collected from PD patients was decomposed into sub-signals via EEMD to replace the conventional filtering techniques. A convolutional layer automatically extracted key features from these sub-signals to train the deep learning classifier, eliminating the need for manual feature engineering. The proposed method can be generalized to identify tremors from motion signals obtained during daily activities without pre-identified features. The proposed approach accurately extracted Parkinsonian tremors from raw signals across various activities, achieving low root-mean-square error and 94.2 ± 1.1% accuracy in differentiating voluntary and tremulous motions in PD patients.

**Supplementary Information:**

The online version contains supplementary material available at 10.1038/s41598-025-08216-7.

## Introduction

Parkinson’s disease (PD) is a neurodegenerative movement disorder primarily affecting individuals over 50 years old. It is characterized by Parkinsonian tremors, a complex, involuntary oscillatory movements that significantly impact mobility and quality of life^1^. These tremors manifest in various forms during different activities. Understanding the characteristics of Parkinsonian tremors is vital for developing applications such as tremor compensation devices to aid patients in managing their condition. In clinical practice, tremor diagnosis often relies on visual observation and standardized rating scales like the Movement Disorder Society-Unified Parkinson’s Disease Rating Scale (MDS-UPDRS). Although these methods are widely used, they are dependent on clinician expertise and can be subject to variability and subjective interpretation, particularly when differentiating Parkinsonian tremor from other types, such as essential tremor. To study tremors objectively, signals such as hand-orientation data and electromyography (EMG) are commonly collected. These signals contain both voluntary motion components (typically below 3 Hz) and tremulous components (ranging from 3 to 10 Hz)^2^ making accurate separation of the two essential for meaningful analysis and application.

The extraction of the tremulous motion from the raw signal is crucial as it is infeasible to directly measure a pure tremulous motion signal using sensors. One way of extracting tremulous motion data is using digital filters such as band-pass filters with the cut-off frequencies confining the pre-determined tremor frequency band or low pass filters to obtain voluntary motions and subtract from the original signal. Ibrahim et al.^2^ applied a 4th order Butterworth band-pass filter and a low-pass filter to the original sensor signal to extract the tremulous and voluntary motions. Also, Widjaja et al.^3^ applied a 3rd order Butterworth high-pass filter with a 1 Hz cut-off frequency to remove the voluntary motion and a 4th order Butterworth low-pass filter with a 15 Hz cut-off frequency to remove the unwanted noise in the accelerometer signal. Extracting the voluntary motion would also benefit tremor suppression application, Xie et al.^4^ proposed an adaptive low pass filter that can adaptively adjust the cut-off frequency based on the patient’s behavior to obtain more accurate extraction result. By applying these filters, the pathological tremor is extracted. However, it is important to note that distinct PD patients and their corresponding activities may exhibit varying tremor characteristics such as frequencies and amplitudes, which can be challenging for the automatic extraction of tremor signals from raw signals. Consequently, a band-pass filter with a narrower cut-off frequency range may not effectively capture the tremulous motion, while using a broader cut-off frequency range could lead to the inclusion of undesired signals. Therefore, a new method to automatically differentiate tremulous and voluntary motions is essential. Automating this differentiation process can support clinicians further by providing more objective and consistent assessments of tremor and enhancing the effectiveness of assistive technologies that rely on accurate detection of tremor from sensor reading prior to tremor compensation.

### Contributions

Existing tremor differentiation methods often rely on manual feature engineering and fixed criteria such as predefined cut-off frequencies, which may limit their ability to capture the heterogeneous nature of tremor across patients and activities. These approaches struggle with generalizability and adaptability when faced with varying tremor amplitudes and frequencies in real-world settings. To overcome these limitations, we propose a method that eliminates the need for handcrafted features and fixed thresholds. The proposed framework combines EEMD and a convolutional bi-directional LSTM to differentiate between voluntary and tremulous motions using raw sensor signals. EEMD decomposes the raw sensor signal into multiple sub-signals, which are then processed by the convolutional layer to extract meaningful features for classification. The model is trained and evaluated on hand-arm orientation data collected from PD patients during diverse activities that exhibit varying tremor characteristics. A figure illustrating the key motivations and contributions of this study is provided in Supplementary Information 1. The main contributions of this work are summarized as follows:

1. A data-driven approach is developed to automatically extract tremor features without relying on manual design or fixed frequency assumptions.
2. EEMD enables adaptive decomposition of raw motion signals to handle tremor variability across different patients and activities.
3. The convolutional bi-directional LSTM architecture is used to classify tremor from unprocessed orientation data effectively.
4. The method demonstrates robust performance across activity-induced variations in tremor, supporting its applicability to real-world PD monitoring scenarios.

### Literature review

Previous works have explored several techniques to separate voluntary and tremulous motion data, such as the signal decomposition algorithms. Wang et al.^5^ applied fast Fourier transform (FFT) to extract the tremulous motion data from the raw signal. Yoon et al.^6^ applied empirical mode decomposition (EMD) to decompose the raw signal into a set of sub-signals for tremor prediction. Furthermore, several works demonstrated the ability of EMD to extract the tremulous motion and its key features^6–8^. Nonetheless, relying solely on signal decomposition algorithms proves insufficient, as they merely decompose the raw signal into sub-signals without the capacity to decide to differentiate the sub-signal types. Aljalal et al.^9^ and Zhang et al.^10^ demonstrated another way of utilize the decomposition method, whereby combining it with machine learning (ML) or deep learning (DL) models to perform classification or differentiation. Therefore, the incorporation of ML or DL models becomes imperative for their accurate differentiation.

To incorporate the ML or DL models in differentiating the tremulous and voluntary motions, several features need to be extracted from the input raw signal for the models to learn the key characteristics of tremors. Rissanen et al.^11^ showed that with the features of time-series signals, the K-means clustering algorithm achieves good differentiation of PD patients and healthy control (HC) subjects, which indicates that K-means clustering is effective in detecting Parkinson’s disease. For supervised classifiers such as support vector machines (SVM), several researchers have proven their ability to classify PD and HC^8,12,13^. Moreover, other supervised classifiers have demonstrated their capabilities in classifying PD and HC, such as the decision tree, Naïve Bayes classifier, K-nearest neighbors (KNN), and linear discriminant analysis^9,14–17^. Besides ML classifiers, several DL classifiers such as artificial neural networks and probabilistic neural networks have also proven their capability to classify the PD and HC with the aid of the feature extraction method^8,18,19^. Lee and Lim^20^ applied a neural network with weighted fuzzy membership functions to classify the PD and HC. However, conventional ML and some DL classifiers used manual feature engineering before performing the training and classification, and hence, a lack of professional knowledge of the investigated signal may lead to poor classification accuracy.

DL classifiers such as convolutional neural networks (CNN) eliminate the need for manual feature extraction. This is due to their multi-layered structure comprising interconnected neurons that autonomously acquire features from the input signal. Hathaliya et al.^21^ proposed a two-dimensional CNN-based classifier with 7 hidden layers and used the accelerometer signal as an input to classify the PD and HC subjects. Additionally, Oktay and Kocer^22^ proposed a convolutional LSTM network that takes the three-dimensional landmarks points of hands as the input to the network to differentiate Parkinsonian tremor and essential tremor. Moreover, Göker^23^ proved that DL classifiers are more effective than ML classifiers by comparing the performances of bi-directional LSTM with SVM, random forest, and KNN in PD detection using power spectral density value. Furthermore, Zhang et al.^10^ have carried out investigations on the capabilities of DL models such as proposing deep residual shrinkage network in classifying PD, HC, and REM sleep disorders. Existing studies in this area have primarily focused on differentiating between PD and healthy individuals, as well as distinguishing various forms of pathological tremors such as essential tremors and Parkinsonian tremors. Nevertheless, the differentiation or classification of Parkinsonian tremors and voluntary motion within a mixed raw signal has yet to receive significant attention. Furthermore, these studies have heavily relied on open-source datasets, in which the measurement details are not readily available.

## Methodology

Developing real-time tremor applications, such as tremor suppression systems for rehabilitative devices, necessitates the accurate extraction of tremulous motion from raw sensor signals. The EEMD is capable of accurately extracting PD patients’ signal characteristics by decomposing raw sensor data into meaningful sub-signals. Then, DL models can be used to differentiate tremulous-containing sub-signals from voluntary motions. In this study, we develop a new architecture that incorporates EEMD and a DL model, namely convolutional bi-directional LSTM, for distinguishing tremulous and voluntary motion in raw sensor signals. A flowchart of the proposed method is provided in Supplementary Information 1.

**Fig. 1:**
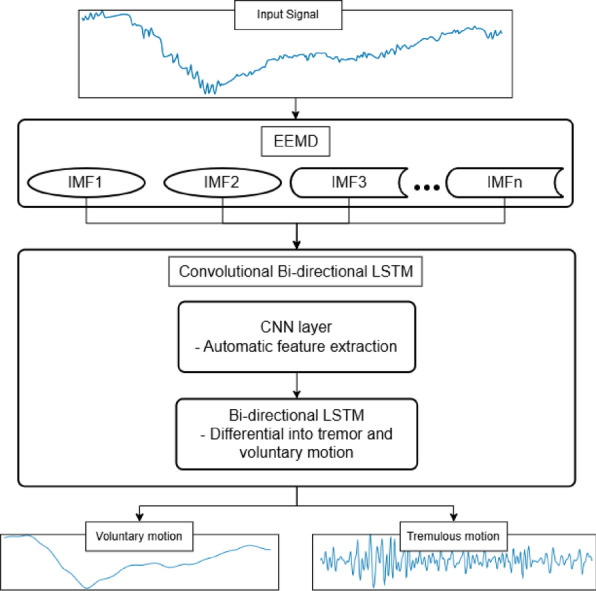
Framework of the proposed method for automatic differentiation of voluntary and tremulous motions in PD patients using the EEMD and convolutional bi-directional LSTM.

The overall framework of the proposed method is demonstrated in Fig. 1. Initially, the raw hand-arm orientation data from PD patients are preprocessed using interpolation and detrending before decomposition. Subsequently, the preprocessed raw data undergo decomposition using EEMD to yield sets of intrinsic mode functions (IMFs). Then, the zero padding and windowing techniques are applied to each IMF, enabling the DL model to carry out differentiation. The convolutional layer of the DL model automatically extracted features from the IMFs time series data, and the bi-directional LSTM layer utilizes these features to differentiate the IMFs time series data into voluntary and tremulous motions.

The proposed method differs from those in the literature as it does not require manual feature engineering; this is automatically performed by the convolutional layer in the proposed convolutional bi-directional LSTM. In contrast, most existing work decomposes the input signal and extracts relevant features from the decomposed signal for the classification task at a later stage^8^. However, this step has been eliminated in our proposed framework. Additionally, most AI applications related to PD focus on the classification of disease or tremor type, with limited work on tremor-voluntary motion classification. Hence, the proposed method provides an alternative approach to extracting tremor from the unprocessed signal without the need for prior analysis to perform manual feature engineering.

### Data acquisition

The clinical study, with protocol no. NMRR-14–1694-21740 [IIR], was conducted at the Neurology Clinic of Penang General Hospital (Malaysia) to acquire data from PD patients. It was approved by the Medical Research Ethics Committee, Secretariat of the National Institutes of Health, Malaysia, and strictly adhered to the ethical guidelines. All participants provided written informed consent.

Three-dimensional orientation data for the lower arm, upper arm, and palm were recorded using the SBG IG-500 A attitude and headings reference system (AHRS) developed by SBG Systems in Rueil-Malmaison, France. The sampling frequency was 100 Hz, and data acquisition was facilitated by Laboratory Virtual Instrument Engineering Workbench (LabVIEW™) software, version 15.0.0, from National Instruments Corporation, Austin, Texas. More information about the software is available at: https://www.ni.com/en-us/support/downloads/software-products/download.labview.html.

Hand-arm motions were recorded for roll, pitch, and yaw at each time instance. Participants performed four distinct actions: resting the upper limbs on the armrests (resting), outstretching the upper limb with fingers separated in front of the chest (outstretching), holding the upper limb in a wing position (wing), and drinking water by taking a cup and then returning the cup to its original position (drinking). Each action lasts 10 s, and the duration of drinking depends on the subject to complete four drinking cycles.

The wing posture and drinking action were executed following the protocol of the Washington Heights-Inwood Genetic Study of Essential Tremor Rating Scale. The remaining actions, upper limb resting and outstretching, were performed according to the protocols outlined by Movement Disorder Society Unified Parkinson’s Disease Rating Scale, with permission from the International Parkinson and Movement Disorder Society.

The study involved 60 PD patients, aged between 50 and 86 years, with a mean age of 70 ± 8.1 years. Among the participants, approximately 60.9% were male and 39% were female. When available, disease severity was assessed using the Hoehn and Yahr staging scale, with stages I to IV represented. Specifically, 16, 22, 12, and 6 patients were categorized into stages I, II, III, and IV, respectively. Most participants receiving tremor-suppressing medication were treated with levodopa—either alone (7 cases) or in combination with other agents such as anticholinergic drugs (2 cases), Trivastal (1 case), or Madopar with Entacapone (1 case). One participant reported taking medication without specifying the type.

Participants were included if they were 50 years of age or older and had a clinical diagnosis of idiopathic Parkinson’s disease, confirmed according to the UK Parkinson’s Disease Society Brain Bank criteria. Exclusion criteria included the use of substances or medications (except prescribed PD treatments) that could affect tremor activity, as well as any underlying medical conditions known to induce tremors.

The tremor ratings were predicted from the static linear regression relating measurement readings with doctors’ observational ratings done in previous work^24^. The predicted tremor rating for each action is as follows. The types of rating scale predicted depend on the action, as aforementioned.

- Resting (Average ± standard deviation, SD): 0.8 ± 0.9.
- Outstretching (Average ± SD): 0.8 ± 0.8.
- Drinking (Average ± SD): 0.4 ± 0.3.
- Wing (Average ± SD): 0.6 ± 0.5.

The raw data from 60 PD patients, obtained from the clinical study, were used for the analysis in this work. The raw data consists of voluntary and tremulous hand-arm orientation data, where the voluntary movement occurs below 3 Hz and the tremulous movement occurs between 3 and 10 Hz^24^. Hand orientation data was chosen over other potential signals, such as EMG, because although EMG can be used to quantify tremorous motion, it is less commonly employed than motion signals^25^. EMG requires more precise sensor placement, making it less practical for wearable devices and challenging to use in real-time applications. Additionally, EMG signals are susceptible to muscle crosstalk, which necessitates extensive signal processing before they can be meaningfully analyzed^26,27^. To prepare the raw sensor data for decomposition, pre-processing steps such as piecewise cubic Hermite interpolating polynomial and the linear detrend using *scipy.signal.detrend* from Python had been performed. These pre-processing steps help capture missing data and smooth the sensor data for accurate analysis.

### Ensemble empirical mode decomposition (EEMD)

After interpolating the raw sensor data, signal decomposition was performed, and the complex raw signal was decomposed into multiple physically meaningful sub-signals known as IMFs. In previous studies, De Lima et al.^28^ had shown the capability of EMD in extracting the tremulous motion; however, the EMD also brings serious issues during the decomposition process, namely the mode-mixing problem. To overcome this issue, two signal decomposition methods, namely EMD and EEMD, were employed, and their performance was systematically compared. The objective is to show that the chosen signal decomposition method is capable of accurately extracting tremulous motion from the raw signal.

EMD is an adaptive and effective decomposition method, which is widely used in decomposing non-stationary and non-linear signals^29^. It extracts a series of IMFs from the input signal by shifting process. An IMF represents a complement to the simple harmonic function and every IMF must satisfy the following criteria: (a) the number of zero crossings and extrema must differ at most by one and, (b) there must be zero in the mean value of the envelope defined by the local extrema at any point. With these criteria, the raw signal can be decomposed with the following steps. First, the local extrema of the input signal $\:\text{x}\left(\text{t}\right)$ are identified$\:.$ These extrema are then connected individually to create upper and lower envelopes, represented as $\:\text{u}\left(\text{t}\right)$ and $\:\text{l}\left(\text{t}\right),$ respectively. The mean of these envelopes $\:\text{m}\left(\text{t}\right)\:$is determined. Subsequently, the temporary IMF$\:{\:h}_{i}\left(t\right)$ is derived using (1) and using the current temporary IMF $\:{h}_{i,j}\left(t\right)$ and previous temporary IMF $\:{h}_{i,j-1}\left(t\right)$ to compute the stoppage criterion $\:{D}_{j}$ as shown in (2). The $\:i,\:\:j$, and $\:T\:$represent the $\:{i}^{th}$ IMF, sifting loop, and the length of signal, respectively.

1 $$\:{h}_{i}\left(t\right)\:=\:x\left(t\right)\:-\:m\left(t\right)$$

2 $$\:{D}_{j}=\frac{\sum\:_{t=0}^{T}{\left|{h}_{i,j-1}\left(t\right)-\:{h}_{i,j}\left(t\right)\right|}^{2}}{\sum\:_{t=0}^{T}{\left|{h}_{i,j-1}\left(t\right)\right|}^{2}}$$

If the stoppage criteria that $\:{D}_{j}$ is smaller than the predefined threshold are satisfied, then the temporary IMF $\:{h}_{i,j}\left(t\right)$ is assigned as new $\:{\text{I}\text{M}\text{F}}_{i}$, else the sifting loop is repeated. After obtaining the new IMF, the new residual $\:r\left(t\right)$ is computed using (3) and the entire process is iterated for this residual.

3 $$\:r\left(t\right)\:=\:x\left(t\right)\:-\:{IMF}_{i}$$

If $\:{\text{I}\text{M}\text{F}}_{i}\:$or $\:r\left(t\right)\:$is lower than a predetermined value, or $\:r\left(t\right)$ becomes a monotone function (contains no more than one extremum), the sifting process is completed, or the last step is repeated. As a result, a sequence of IMFs can be obtained.

Even though EMD can effectively decompose the complex non-stationary and non-linear signal into multiple physically meaningful components, it also brings some serious problems during the decomposition process. The mode-mixing problem is the significant drawback brought by the EMD where different frequency components may coexist in the same IMF^30^.

To eliminate the mode-mixing phenomenon and obtain the actual process of the input signal, a new noise-assisted signal analysis method proposed by Wu and Huang^31^ which is EEMD, is employed. The process involves multiple steps. First, a white noise series $\:{w}^{n}\left(t\right)$ is added to the original signal$\:{\:x}^{n}\left(t\right)$ as

4 $$\:{x}^{n}\left(t\right)=\:x\left(t\right)+{w}^{n}\left(t\right)$$

where $\:n$ = 1, 2, 3, …, N represents different white noise series and N denotes the number of ensembles. Subsequently, the signal with added noise is decomposed using EMD. This leads to the extraction of IMF components from the decomposed signal. To enhance accuracy, the entire process is repeated multiple times with different white noise series. The outcome is the ensemble mean of the residue components and the IMFs obtained from these decompositions.

### Classification

Recall that automatic differentiation/classification of tremulous and voluntary motions is crucial for assistive technology such as active tremor suppression. To automate the differentiation process from the IMFs, DL was adopted to learn their key characteristics.

#### Training and testing data

In this study, hand-arm orientation data from 60 PD patients are analyzed. The training dataset consists of 48 PD patients who were selected at random, each of whom completed 4 different activities, was measured on 3 distinct body parts and 3 axes, and had an average of 8 IMFs. On the other hand, the remaining 12 PD patients engaged in 4 distinct activities, across 3 different body parts and 3 axes, with an average of 8 IMFs considered for the testing dataset.

To perform time series classification using DL classifiers, it is essential to standardize the time series length for consistent input to the network. To achieve this, the non-overlapping windowing technique with a window size of 50 data lengths was applied to every IMF. Additionally, the process involved checking the length of the last window for each IMF. If the length was less than 50 data points, the zero-padding technique was applied at the end of the window to maintain consistency. Following the application of padding and windowing techniques to the training and testing datasets, a total of 182,368 samples were used for training, and 45,594 samples were used for testing the DL models.

To provide a robust estimates of the model’s performance, a re-training procedure is applied, whereby the whole dataset is shuffled before each time the training procedure initiates and split the data into training and testing datasets with an 80:20 ratio. Utilizing those datasets, 10 times of training are done, and all the models are saved for evaluation later.

#### Convolutional bi-directional LSTM

**Fig. 2:**
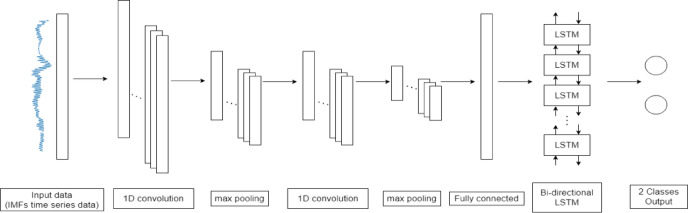
Architecture of the proposed convolutional bi-directional LSTM.

This study proposes a DL classifier model called the convolutional bi-directional LSTM. The model employs a CNN in the first phase to automatically extract features from IMFs time series data. The extracted features are then fed to a bi-directional LSTM layer for automatic differentiation. Figure 2 shows the architecture of the convolutional bi-directional LSTM.

The model consists of several key hyperparameters. The first convolutional layer employs 30 filters with a kernel size of 20 and stride of 1 and applies the rectified linear unit (ReLU) activation function. This is followed by a max-pooling layer with a pool size of 2. The second convolutional layer also uses 30 filters with a smaller kernel size of 10 and a stride of 1 and the ReLU is used as the activation function. Another max-pooling layer with a pool size of 2 follows this. The fully connected layer has a single unit and utilizes the ReLU activation function.

**Fig. 3:**
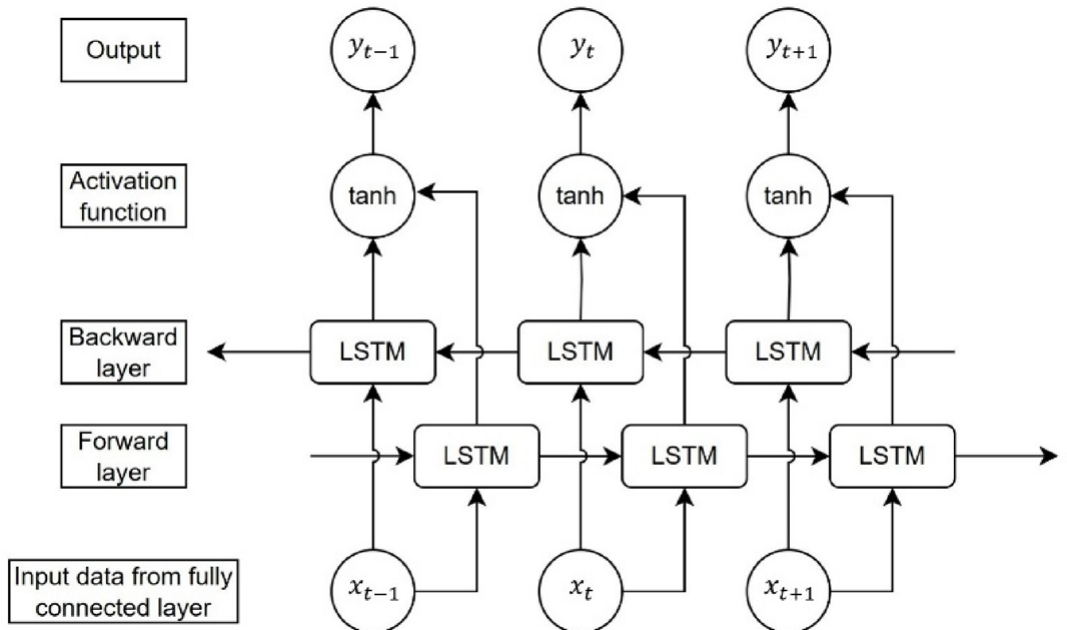
Architecture of Bi-directional LSTM layer.

Following the fully connected layer, the output sequence proceeds into the bi-directional LSTM layer, as illustrated in Fig. 3. This layer comprises both forward and backward LSTM units, each hosting 64 LSTM cells. The number of LSTM cells was tested incrementally from 1 to 100. When the number of LSTM cells reached 60, the performance of the model began to converge. Therefore, 64 LSTM cells were chosen for the final model. This design choice ensures efficient handling of lengthy input sequences and affords ample model complexity to discern intricate patterns and relationships within the data. The structure of the LSTM cell, provided in Supplementary Information 1, incorporates three pivotal gates: a forgetting gate, an input gate, and an output gate, which collectively facilitate learning the dependencies inherent in the input data.

Subsequently, the output of each LSTM cell undergoes a final tanh activation function before transitioning to the next layer. Concluding the architecture is a single-unit output layer employing a sigmoid activation function. For optimization, the stochastic gradient descent (SGD) is employed with a modest learning rate of 0.01. During training, the model is guided by the binary cross-entropy loss function over a span of 100 epochs, with a minimum batch size set to 64.

In this study, the performance of the proposed DL model is compared with the convolutional LSTM presented in the work by Oktay and Kocer^22^ using the same training and testing data. The convolutional LSTM network consists of two components, the first component is CNN, which is built for feature extraction purposes, and the second component is an LSTM network that is used for classification^22^. Furthermore, the investigation extends to include standard ML classifiers such as KNN, and Naïve Bayes, offering a comprehensive comparison with the proposed DL model. The hyperparameter configurations for each classifier are outlined below:

- KNN: Employing the Euclidean distance metric, the classifier is configured with a neighbourhood size of 3.
- Naïve Bayes: Utilizing Gaussian Naïve Bayes, this classifier operates under the assumption of feature independence.

For a comprehensive understanding of the convolutional LSTM, KNN, and Naïve Bayes models, a detailed description is provided in Supplementary Information 2. In addition, manual feature engineering is required for the training of the KNN and Naïve Bayes classifiers. The optimal features were selected through a trial-and-error process. Initially, candidate features were identified based on the literature review. These features were then extracted from the decomposed IMFs and statistically evaluated to determine their effectiveness in distinguishing between tremulous and voluntary motions. As a result, four features were selected: instantaneous frequency^32^kurtosis^12^peak magnitude divided by RMS value^16^and sample entropy. These features were found to best capture the characteristics of tremorous and voluntary motions and demonstrated a significant impact on the performance of the machine learning models. Detailed descriptions of these features are provided in Supplementary Information 2.

#### Performance evaluation metrics

To evaluate the efficacy of signal decomposition techniques, several combinations of IMFs are compared against the most used methods for signal differentiation, namely band-pass and low-pass filters. A 4th-order Butterworth band-pass filter with a cut-off frequency of 3 to 10 Hz is used to extract the tremulous motion, while a low-pass filter with a cut-off frequency of 3 Hz is used to extract the voluntary motion. Furthermore, the logged root-mean-square error (log(RMSE))is used to quantify the performance of the decomposition methods.

5 $$\:{log}\left(RMSE\right)={{log}}_{10}\sqrt{\frac{1}{n}\sum\:_{i=1}^{n}{\left({x}_{i}-{\widehat{x}}_{i}\right)}^{2}}$$

where $\:{x}_{i},\:{\widehat{x}}_{i}$ is the benchmarked signal and extracted signal, respectively, while n is the number of data points of the benchmarked signal. For the differentiation, the performances of five models are evaluated based on the accuracy, precision, sensitivity, specificity, F1 score, and area under the Receiver Operating Characteristic Curve (AUC-ROC) given as

6 $$\:Accuracy=\:\frac{TP+TN}{TP+TN+FP+FN}$$

7 $$\:Sensitivity=\frac{TP}{TP+FN}$$

8 $$\:Specificity=\frac{TN}{TN+FP}$$

9 $$\:Precision=\frac{TP}{TP+FP}$$

10 $$\:F1\:Score=2\times\:\frac{Precision\times\:Sensitivity}{Precision+Sensitivity}$$

where TP (true positive) is the number of actual cases of tremulous motion correctly identified by the models as tremulous motion, TN (true negative) is the number of actual cases of voluntary motion correctly classified as voluntary motion, FP (false positive) is the number of cases of voluntary motion wrongly recognized as tremulous motion and FN (false negative) is the number of cases of tremulous motion wrongly distinguished as voluntary motion.

#### Code availability

The code used to implement the proposed model and analysis is openly available on Zenodo at 10.5281/zenodo.15582786. This archived version ensures reproducibility and long-term accessibility of the software used in this study. The dataset used and/or analyzed during the current study is available from the corresponding author upon reasonable request.

## Results

The simulations of the proposed approach were performed using Python 3.10.12 in Google Colab with hardware specification of Intel Xeon CPU with 2 vCPUs (virtual CPUs) and 13GB of RAM. To evaluate the effectiveness of the proposed approach, EMD and EEMD were tested on the signals of four activities from 60 patients. The performance of decomposition methods was compared using the $\:\text{log}\left(\text{R}\text{M}\text{S}\text{E}\right)\:$score, with filtered tremulous and voluntary motion as benchmarks. In this section, the simulation results were represented as the $\:\text{log}\left(\text{R}\text{M}\text{S}\text{E}\right)\:$score of five patients, and the datasets that were tested from the roll of the lower arm were used to illustrate the performance of the signal decomposition methods. Displayed in Table 1 were the performance results of various combinations of IMFs denoted for both EMD and EEMD, in terms of average $\:\text{log}\left(\text{R}\text{M}\text{S}\text{E}\right)\:$score for each activity among five different PD patients.

From Table 1, it is important to note that a single IMF (such as IMF1 or IMF2) did not represent well the intricate details of tremulous motion; instead, it captured only a portion of this motion. Hence, to achieve accurate extraction of both tremulous and voluntary motions, combinations of various IMFs were necessary. Also, it was observed that the IMF12 derived from EEMD had a better performance, as shown by the lowest error for most activities. Although the IMF2 had a lower RMSE score compared to IMF12 in the drinking activity, and the difference between IMF2 and IMF12 was small, IMF12 could decompose better for various types of activities, such as resting, outstretching, and wing.

**Fig. 4:**
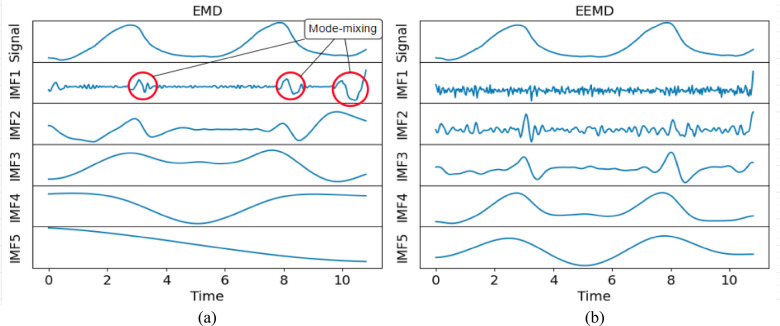
Sample decomposition performance of (**a**) EMD and (**b**) EEMD for drinking activity. The y-axis is the roll (deg).

**Fig. 5:**
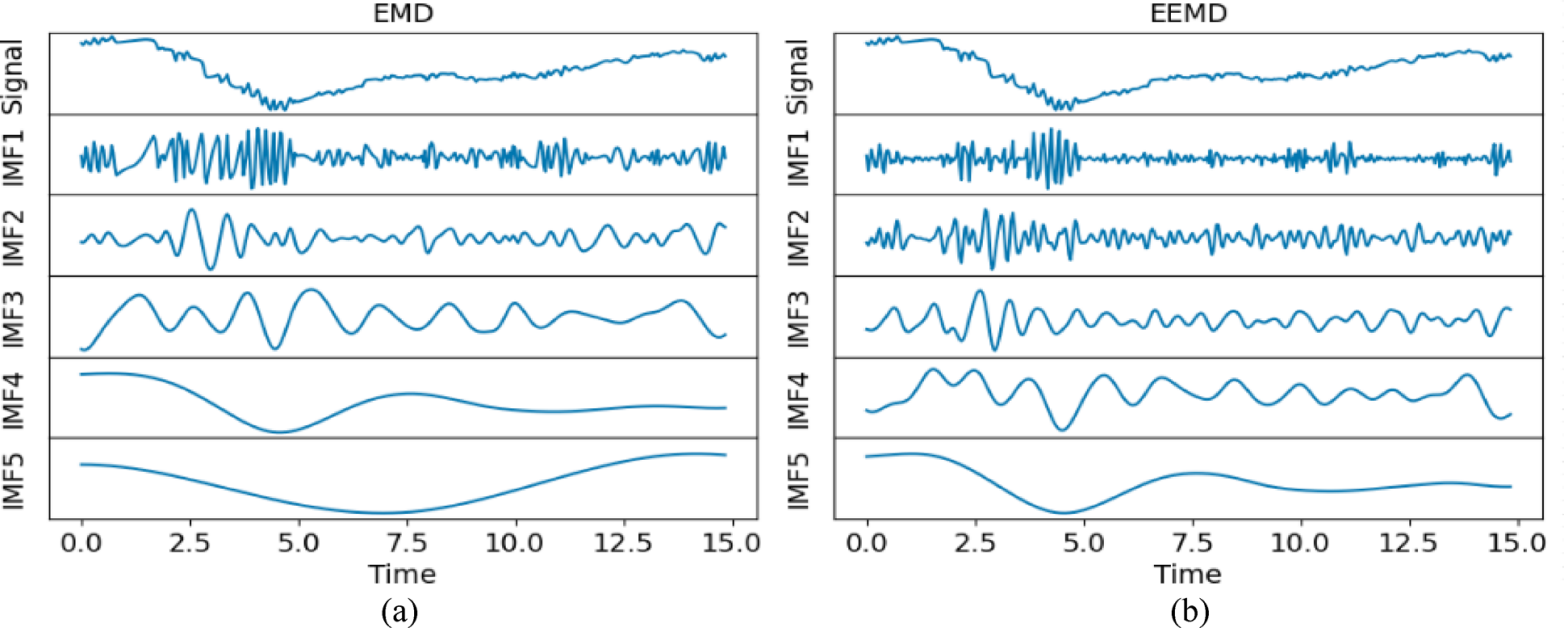
Sample decomposition performance of (**a**) EMD and (**b**) EEMD for outstretching activity. The y-axis is the roll (deg).

**Fig. 6:**
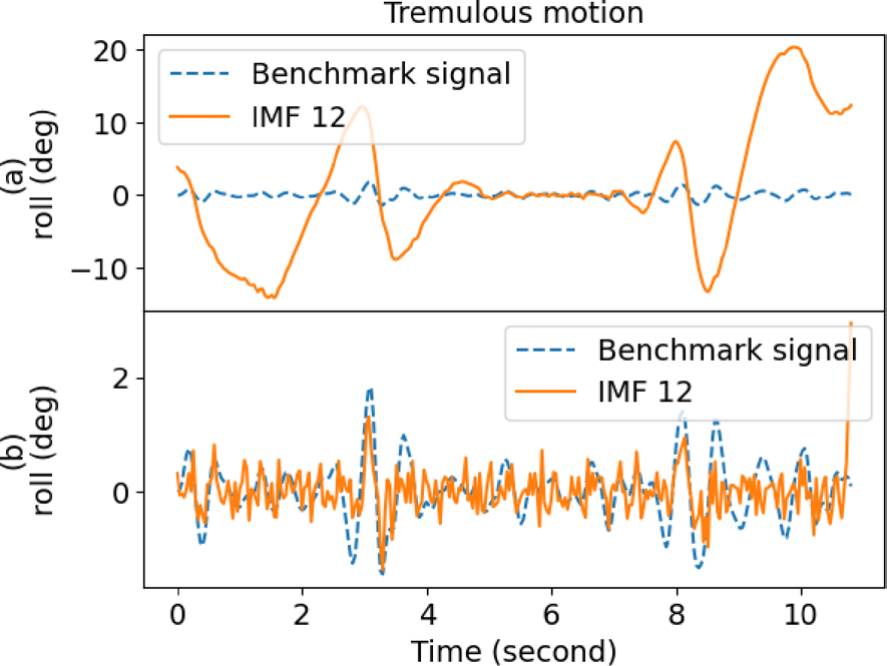
Sample tremulous motion comparison of (**a**) EMD and (**b**) EEMD for drinking activity.

**Fig. 7:**
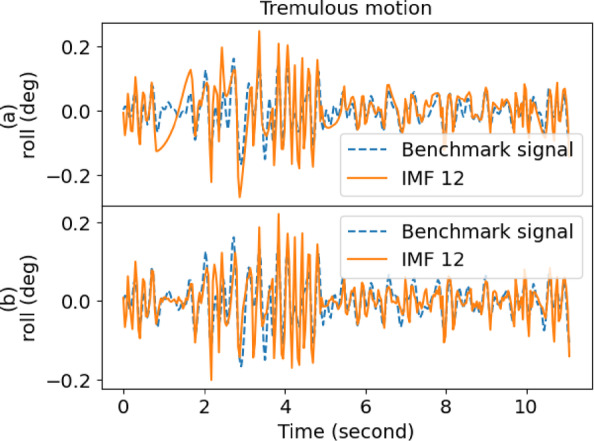
Sample tremulous motion comparison of (**a**) EMD and (**b**) EEMD for outstretching activity.

**Fig. 8:**
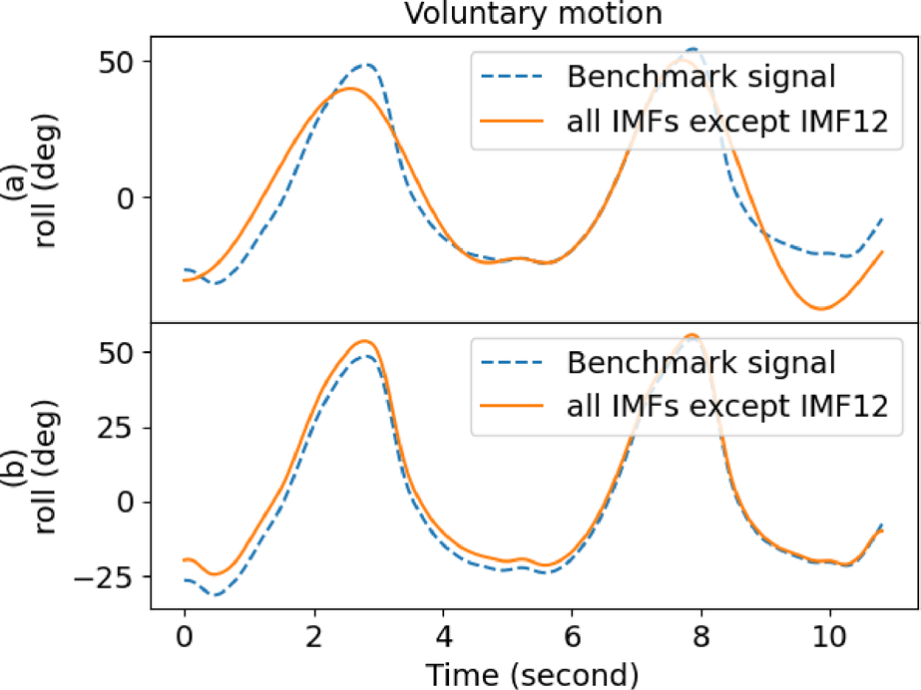
Sample voluntary motion comparison of (**a**) EMD and (**b**) EEMD for drinking activity.

**Fig. 9:**
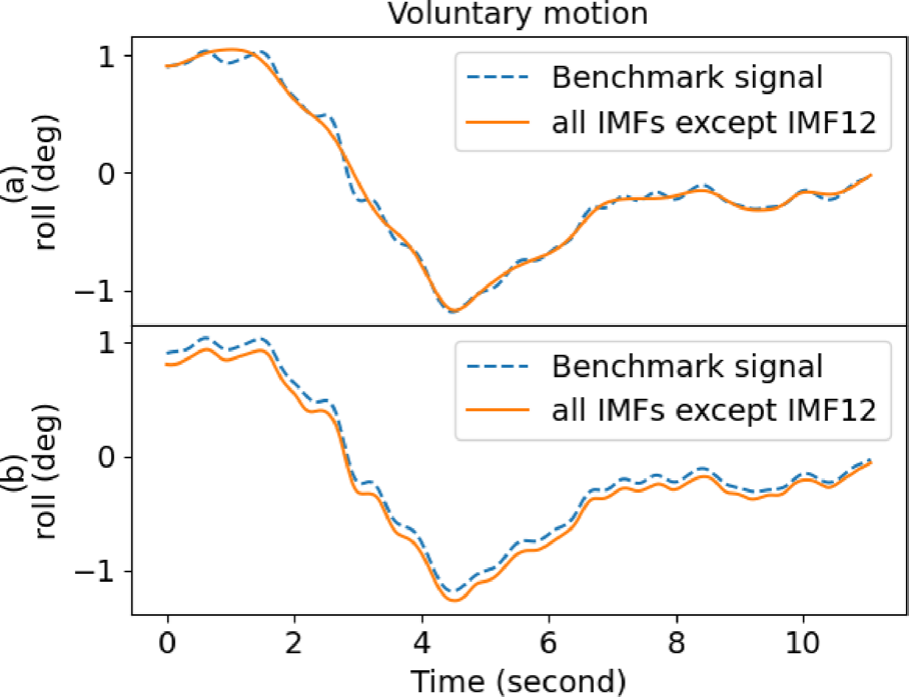
Sample voluntary motion comparison of (**a**) EMD and (**b**) EEMD for outstretching activity.

Table 1 also gives the performance of EMD, where it yielded a low score in decomposing the raw signal. However, IMF12 had the lowest score for outstretching and wing, whereas IMF1 was the best for drinking and resting. As a result, the performance of EMD was inconsistent and fell short of expectations. Moreover, the overall performance of EEMD was better than EMD when comparing the scores. Figures 4 and 5 show the sample comparison between EMD and EEMD using drinking and outstretching signals, respectively. Figures 6 and 7 show the comparison between EMD and EEMD in extracting tremulous motion using drinking and outstretching signals, respectively. Figures 8 and 9 show the comparison between EMD and EEMD in extracting voluntary motion using drinking and outstretching signals, respectively. Further elaborations on the results in Figs. 4, 5, 6, 7, 8 and 9 are provided in the discussion section.

In summary, the EMD performed well in capturing and representing the tremulous motion for certain activities, such as resting and outstretching activities. However, the strength of EMD could not be guaranteed, as the mode-mixing problem shown in Fig. 4 might occur when EMD decomposes activities like drinking, causing the tremulous motion not to be extracted accurately. On the other hand, EEMD outperformed EMD in terms of RMSE score, and it also reduced the mode-mixing problem brought by EMD, demonstrating its robustness and effectiveness in extracting and representing the tremulous motion from the raw signal. Consequently, EEMD was more suitable for signal decomposition, and the combination of IMF12 produced by EEMD served as the tremulous combination, while the remaining IMFs served as the voluntary combination.

**Table 1:** Performance of EMD and EEMD for extraction of tremulous motion. IMFxy denotes the combination of IMFx and imfy. $\:\text{log}\left(\text{R}\text{M}\text{S}\text{E}\right)$ score with * indicates the lowest score in the activity.

Decomposition	IMF	Activity			
method		Drinking	Resting	Outstretching	Wing
EMD	IMF1	−0.72*	−1.47*	−1.82	−1.15
	IMF2	0.31	−1.25	−1.82	−1.05
	IMF12	0.30	−1.45	−1.84*	−1.23*
	IMF23	0.96	−1.16	−1.58	−0.97
EEMD	IMF1	−1.65	−2.69	−2.53	−2.65
	IMF2	−1.73*	−2.84	−2.53	−2.52
	IMF12	−1.72	−2.86*	−2.64*	−2.68*
	IMF23	−1.48	−2.62	−2.23	−2.21

Using EEMD, the IMFs of 48 patients performing four different activities were extracted and preprocessed to be the training data for DL and ML classifiers. The DL classifier took the IMFs as input data while ML classifiers took the four features extracted from the IMFs as input data. Additionally, note that all training data were labelled using the same criterion, whereby tremulous features or IMF windows were designated as positive, and voluntary features or IMF windows were designated as negative. As aforementioned, the re-training procedure was applied to the training process. After the training process, the performance of each model was evaluated using the same unseen testing dataset, which consisted of 45,594 samples for each DL and ML. The results are presented in Table 2. Table 2 presents the average performance metrics of five different models. The proposed convolutional bi-directional LSTM demonstrates impressive performance, with an accuracy of 94.2% ± 1.1%, precision of 0.96 ± 0.09, sensitivity of 0.78 ± 0.09, specificity of 0.98 ± 0.04, an F1 score of 0.85 ± 0.03, and an AUC-ROC of 0.99 ± 0.003. Notably, it outperforms the convolutional LSTM, which also requires IMF as input data and performs automatic feature extraction. These results underscore the robustness and effectiveness of the convolutional bi-directional LSTM in both recognizing underlying patterns and accurately classifying tremulous and voluntary motions.

When comparing the performance of ML classifiers, both KNN and Naïve Bayes exhibited notable performance, highlighting their effectiveness in classifying tremulous and voluntary motions when manual feature extraction is required. However, it is important to note that the performance of ML classifiers benefited from a prior analysis of the input IMF to identify key features of tremulous and voluntary motion. Consequently, the performance of ML classifiers cannot be directly compared with the proposed convolutional bi-directional LSTM.

This comparison aims to assess the proposed model’s accuracy without any in-depth analysis of the input IMF, highlighting its capability to recognize underlying patterns in various input data. Despite not relying on prior manual feature extraction, the proposed convolutional bi-directional LSTM still achieves a notable accuracy of 94.2% ± 1.1%. This indicates that the proposed model can automatically learn and extract relevant features through multiple layers of processing, demonstrating its robustness and efficacy in real-world applications.

**Table 2:** Performance metric for the classifiers.

	Automatic feature extraction						Manual feature extraction						
(Average $\:\pm\:$ SD)	Convolutional bi-directional LSTM			Convolutional LSTM			KNN			Naïve Bayes			
Accuracy (%)	94.2	$\:\pm\:$	1.1	85.6	$\:\pm\:$	8.9	93.2	$\:\pm\:$	0.3	93.2	$\:\pm\:$	0.3	
Precision	0.96	$\:\pm\:$	0.09	0.68	$\:\pm\:$	0.47	0.86	$\:\pm\:$	0.01	0.86	$\:\pm\:$	0.01	
Sensitivity	0.78	$\:\pm\:$	0.09	0.43	$\:\pm\:$	0.37	0.89	$\:\pm\:$	0.01	0.89	$\:\pm\:$	0.01	
Specificity	0.98	$\:\pm\:$	0.04	0.99	$\:\pm\:$	0.01	0.95	$\:\pm\:$	0.01	0.95	$\:\pm\:$	0.01	
F1 score	0.85	$\:\pm\:$	0.03	0.5	$\:\pm\:$	0.39	0.87	$\:\pm\:$	0	0.87	$\:\pm\:$	0	
AUC-ROC	0.99	$\:\pm\:$	0.005	0.78	$\:\pm\:$	0.22	0.92	$\:\pm\:$	0.01	0.92	$\:\pm\:$	0.01	

## Discussion

This study introduces a novel approach to the automatic extraction and differentiation of tremulous and voluntary motions from raw signals associated with PD. Existing literature commonly employs filtering and decomposition algorithms, such as adaptive band-pass filters, weighted Fourier transforms, and bandlimited multiple Fourier linear combiners to extract tremors^33–35^. However, these methods often require detailed and accurate models for the system, and a lack of prior knowledge of the characteristics of the input signal and the associate noise will reduce the accuracy of these models, limiting their general applicability. In contrast, EMD addresses this issue, and De Lima et al.^28^ explored its potential in effectively separating tremulous and voluntary signals. However, EMD has limitations as it suffers from the mode-mixing problem.

From Figs. 4 and 5, it can be inferred that by using EMD, the decomposed IMF1 still contains components of different frequencies as shown (circle) in Fig. 4(a), which indicates the existence of a mode-mixing problem. However, by performing the decomposition using EEMD, the mode-mixing problem has been greatly reduced by the added masking of the white noise signal as illustrated in Fig. 4(b). The mode-mixing problem greatly influences the ability of the decomposition methods to extract the tremulous motion from the raw signal. This problem can be observed in the top graphs in Figs. 6 and 7, which show the comparison of extracted tremulous motion with the benchmark signal using EMD and EEMD. The mode-mixing problem causes EMD (Fig. 6(a) and Fig. 7(a)) to have poor decomposition results. Despite the challenge of mode-mixing, EEMD demonstrates better performance in representing voluntary motion, as illustrated in Figs. 8 and 9. In Figs. 8(a) and 9(a), the use of EMD to extract signals reveals significant phase shifts and a noticeable disparity in signal patterns compared to the benchmark signal. Conversely, in Figs. 8(b) and 9(b), using EEMD results in minimal phase shifts and an extracted signal pattern that accurately mirrors the benchmark signal. Therefore, the EEMD can extract and represent the tremulous and voluntary motion accurately and perform well for different activities.

Additionally, it is worth mentioning that the EEMD decomposition yielded an average of 8 IMFs, out of which only IMF1 and IMF2 were represented as tremulous motion. This implied that only 25% of training samples had true labels, while the remaining 75% of training samples were labelled as false. Despite this class imbalance, the convolutional bi-directional LSTM model demonstrated remarkable performance in terms of accuracy, precision, and specificity. These results indicated that the model effectively learned and captured relevant patterns in the data, surpassing the performance of other models.

It is worth noting that the feature analysis for ML classifiers was conducted using filtered tremulous and voluntary motions as benchmarks. As previously mentioned, distinct PD patients and their corresponding activities may exhibit varying tremor characteristics, meaning that the band-pass and low-pass filters with fixed cut-off frequencies may not effectively capture all instances of tremulous motion. However, the proposed convolutional bi-directional LSTM model is designed to learn the key characteristics of tremulous motion dynamically, enabling it to handle various scenarios. This adaptability makes it more suitable for real-world implementation, as it does not rely on fixed filters and can automatically adjust to the unique tremor patterns exhibited by different patients during different activities.

The performance of the proposed convolutional bi-directional LSTM was compared to other studies in the literature, as shown in Table 3. However, most AI model applications related to PD focus on classifying the disease, tremor type, or severity, rather than differentiating between tremulous and voluntary motions. Therefore, a direct comparison is not feasible due to the different nature of the classifications. Nonetheless, examining how well these models perform their specific tasks provides insight into the effectiveness of our proposed model through performance comparison.

From Table 3, it can be observed that the accuracy of models from other studies ranges from 81.25 to 100%. Thus, an accuracy greater than 80% is considered acceptable. The proposed method demonstrates improved performance with an accuracy of 94.2%. It is noteworthy that the studies by Gulay et al.^8^ and Ai et al.^36^ relied on manual feature extraction techniques before model training. For example, Gulay et al.^8^ achieved 100% accuracy using features extracted through EEMD and vector autoregression, followed by principal component analysis to reduce dimensionality and select the most meaningful features. In contrast, the proposed model performs end-to-end learning by automatically extracting features directly from raw data using a convolutional bi-directional LSTM architecture to the decision making of whether it is tremor or non-tremulous action. Despite not relying on manually engineered features, the proposed model still achieves remarkable performance. This demonstrates the strength and practicality of the proposed method for tremulous and voluntary motion classification, especially considering its reduced reliance on manual preprocessing steps, which makes it more scalable and adaptable to real-world applications.

**Table 3:** Comparison of the proposed method with other studies in the literature.

	Classification task	Model used	Performance (Accuracy (%))	
Gulay et al.^8^	Parkinson’s disease classification	1. ANN	ANN	:100
		2. Decision Tree	Decision Tree	:84.38
		3. SVM	SVM	:81.25
		4. KNN	KNN	:81.25
Kim et al.^37^	tremor severity quantification (Classification of UPDRS)	CNN	85	
Ai et al.^36^	Classification of parkinsonian and essential tremor	SVM	88	
Oktay and Kocer^22^	Classification of parkinsonian and essential tremor	Convolutional LSTM	90	
Proposed method	Tremor-voluntary classification	Convolutional bi-directional LSTM	94.2 $\:\pm\:\:$1.1	

In practical applications, the improvement in accuracy provided by the proposed model can be significant, particularly in scenarios where accurate differentiation between tremulous and voluntary movements is critical. Such critical differentiation is required in tremor compensation that only tremor is to be compensated with minimal alteration to the intended motion in the daily activities. To use ML models, such as KNN and Naïve Bayes in practical applications, an extensive feature analysis and selection process is required. This manual process involves identifying, testing, and optimizing features for machine learning, which is labor-intensive and iterative. Selecting the optimal features takes a long time for the analysis to determine the best characteristics of tremulous and voluntary motion across different actions. When tested with suboptimal features, those that do not fully capture the characteristics of the input signal, both the accuracies for KNN and Naïve Bayes dropped significantly to 84.07%. In contrast, the proposed model does not face this issue, as it directly uses the input signal and automatically extracts relevant features, enabling it to effectively distinguish between tremulous and voluntary motion in practical applications.

The automatic feature extraction capability of the proposed model offers substantial real-world benefits compared to the manual feature extraction required by the existing ML models. The feature selection and analysis process for the ML model was time-consuming. In contrast, the proposed model can save significant time by performing feature analysis autonomously. One of the key advantages of deep networks is their ability to eliminate the need for manually extracting handcrafted features; these features are learned during the training process^22^. By leveraging DL, the features distinguishing essential tremor and Parkinsonian tremor are learned directly from the data, without the need for prior feature engineering.

A detailed comparative analysis was conducted to evaluate the accuracy of the proposed model when the patient performs different actions.

**Table 4:** Performance metrics for each action by the proposed model.

Model	Metric	Overall	Resting	Outstretching	Drinking	Wing
Proposed	Accuracy (%)	94.79	85.82	84.78	99.62	85.06
Model	Precision	0.99	1	1	0.99	1
	Sensitivity	0.78	0.48	0.44	0.99	0.45
	Specificity	0.99	1	1	0.99	1
	F1 Score	0.87	0.65	0.61	0.99	0.62
	AUC-ROC	0.99	0.74	0.72	0.99	0.73

From Table 4, it is evident that the proposed model excels in differentiating the drinking action, achieving near-perfect performance in this scenario. This can be attributed to the nature of the drinking task, which involves dynamic, multi-phase voluntary and tremulous movements that often elicit more pronounced tremor signals, particularly kinetic and postural tremors. These more distinct signal patterns are effectively captured by the model, enhancing classification accuracy. In contrast, actions such as resting, outstretching, and winging are relatively static or continuous in posture, where tremor signals tend to be weaker and less distinguishable from baseline motion or sensor noise. As a result, the model’s performance for these actions is slightly lower. Nonetheless, the proposed model demonstrates robust overall performance, particularly in differentiating between tremulous and voluntary motions, as indicated by its high specificity and precision.

Moreover, computational time is crucial for implementing the proposed approach in real-time applications. In comparison to existing literature, for instance, Tang et al.^38^ developed a real-time RNN-based gait pattern recognition system with an average time lag of 650 ms. Similarly, Borzì et al.^39^’s real-time freeze-of-gait detection using multi-head convolutional neural networks achieved a remarkably short detection time of just 43 ms. These brief computational times highlight the feasibility of these models for real-time applications. In contrast, this study involves an average decomposition time of 6.22 ± 3.22 s and 4 ms per timestep for DL classification.

Consequently, the computational time required by the proposed method poses limitations for seamless integration into real-time scenarios, with the decomposition step notably contributing to the overall processing duration. Therefore, further optimization of the decomposition step is needed to enhance the real-time applicability of the proposed method. Potential strategies include improving the existing EEMD technique or exploring improved variants like complete empirical mode decomposition with adaptive noise. Additionally, investigating alternative decomposition algorithms, such as the wavelet transform, or developing AI models to replace conventional decomposition methods is also being contemplated.

Furthermore, the computational cost of the Convolutional Bidirectional LSTM model was assessed by comparing its training and inference times under different dataset sizes.

**Table 5:** Training and inference time comparison of the proposed model in different sizes of datasets.

	Convolutional bidirectional LSTM		
Dataset Size (Patients)	Training Time (seconds)	Inference Time (seconds)	Ratio (Training/Inference)
20	902	2.79	323.30
60	2548	42.2	60.38
Increment (×)	2.8×	15×	-

From Table 5, the proposed model required a long time in training for both datasets, which is due to the model complexity and the need for more time to learn the characteristics of the input signal. However, this long training time also brings another benefit: it eliminates the need for feature analysis of input data, which often takes a long time to select the optimal features. Furthermore, it should be noted that each patient will have different characteristics in their tremor, resulting in repetitive feature analysis for each patient, whereas the automatic feature extraction of the proposed model has proven its generalizability across different patients. To investigate the practical applicability of the proposed model, the inference time demonstrates that it performs well with 20 patients, requiring only 2.79 s to differentiate between tremulous and voluntary motions. When the dataset increased to 60 patients, the inference time also increased but remained under 1 min for completing the differentiation.

## Conclusion

In this study, a model was developed to automatically differentiate between voluntary motion and Parkinsonian tremor using raw non-labelled hand-arm orientation data from 60 PD patients. The combination of EEMD and convolutional bi-directional LSTM was employed. Two decomposition algorithms, namely EMD and EEMD, were compared. While EMD performed well in certain activities, it faced challenges in others due to mode-mixing issues, whereas EEMD proved to be a more reliable and effective choice. With low $\:\text{log}\left(\text{R}\text{M}\text{S}\text{E}\right)\:$scores and the ability to mitigate mode-mixing problems, EEMD emerged as the preferred method for decomposing raw signals into voluntary and tremulous motions, demonstrating its capability to replace traditional filtering techniques. With the use of decomposed IMFs, the convolutional bi-directional LSTM model performed well with accuracy of 94.2 ± 1.1%, showcasing its accurate automatic feature extraction and differentiation capabilities. This outcome demonstrates the effectiveness of the proposed approach in differentiating voluntary and tremulous motion in PD patients without filtering and manual features engineering; however, there is still room for improvement in the computational time of the proposed method, which may limit its implementation in real-time applications. Therefore, future work should focus on extending this study by implementing the proposed approach in real-time and exploring more efficient signal decomposition methods or replacing them with AI algorithms to reduce computational time.

## Electronic supplementary material

Below is the link to the electronic supplementary material.

Supplementary Material 1

Supplementary Material 2

## Data Availability

The dataset used and/or analyzed during the current study is available from the corresponding author upon reasonable request.
