# Supplementary material for: Automatic differentiation of voluntary and tremulous motion using ensemble empirical mode decomposition and convolutional Bi-directional LSTM
Source: Sci Rep. 2025 Oct 8;15:35064. doi: 10.1038/s41598-025-08216-7 (PMC12508208; doi:10.1038/s41598-025-08216-7)
Supplement: Supplementary file 1 — Supplementary Material 1 [file 41598_2025_8216_MOESM1_ESM.docx]

**
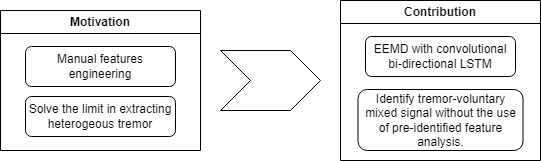
**

**Fig. A**. **1** Motivation and Contribution of this study.


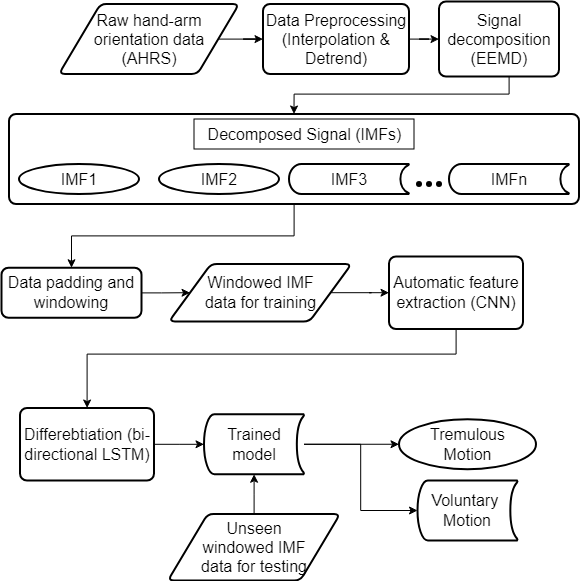


**Fig. A. 2** Flowchart of the proposed method for automatic differentiation of voluntary and tremulous motions in PD patients using the EEMD and convolutional bi-directional LSTM.


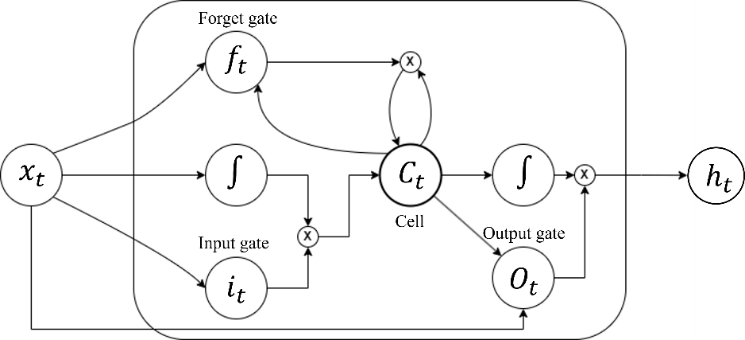


**Fig. A. 3** Architecture of LSTM cell.
