# Supplementary material for: Automatic differentiation of voluntary and tremulous motion using ensemble empirical mode decomposition and convolutional Bi-directional LSTM
Source: Sci Rep. 2025 Oct 8;15:35064. doi: 10.1038/s41598-025-08216-7 (PMC12508208; doi:10.1038/s41598-025-08216-7)
Supplement: Supplementary file 2 — Supplementary Material 2 [file 41598_2025_8216_MOESM2_ESM.docx]

## Features

Feature extraction is crucial for an ML classifier to learn the essential characteristics of the input signal. This process involves extracting four key features from the IMF signal, such as instantaneous frequencies, kurtosis, peak-to-RMS ratio, and sample entropy. These features were fed into the ML classifiers, e.g., SVM, KNN, and Naïve Bayes for further differentiation tasks. A total of 182,368 features were extracted from the training dataset and 45,594 features were extracted from the testing datasets for the training and testing of ML models respectively.

Instantaneous frequency: First, to obtain instantaneous frequency, the Hilbert Huang transform (HHT) should take place. Hilbert Huang transforms consist of two main parts that are EMD and Hilbert transform. As in the previous stage, the raw sensor data had already been decomposed by EEMD and obtain the series of IMFs, thus the Hilbert transform will be performed in each of the IMF obtained in the previous stage.

| $\mathbf{H}\left[ \mathbf{x}\left( \mathbf{t} \right) \right]\mathbf{=y}\left( \mathbf{t} \right)\mathbf{=}\frac{\mathbf{1}}{\boldsymbol{\pi}}\mathbf{p.v.}\int_{\boldsymbol{-\infty}}^{\boldsymbol{\infty}} \frac{\mathbf{x}\left( \boldsymbol{\tau} \right)}{\boldsymbol{(t-\tau)}}\mathbf{du}$ |  |
| --- | --- |

Where $x(t)$ is k^th^ IMF and $p.v.$ indicate the principal value of the singular integral. Thus, the signal can be defined as:

| $\mathbf{z}\left( \mathbf{t} \right)\mathbf{=x}\left( \mathbf{t} \right)\mathbf{+iy}\left( \mathbf{t} \right)\mathbf{=a(t)}\mathbf{e}^{\boldsymbol{i\theta(t)}}$ |  |
| --- | --- |

Therefore, the instantaneous amplitude and phase can be found.

| $a\left( t \right)= \sqrt{x^{2}+y^{2}}$ |  |
| --- | --- |
| $\theta\left( t \right)=arctan(\frac{y}{x})$ |  |

With the instantaneous phase angle, instantaneous frequency can be found.

| $\omega\left( t \right)=\frac{d\theta(t)}{\mathrm{dt}}$ |
| --- |

To quantify the difference between tremulous and voluntary motion based on their instantaneous frequency, a tremulous frequency counter can be employed. This counter calculates the number of instantaneous frequencies that fall within the range of 3 to 12 Hz, which corresponds to the frequency range of tremulous motion. By using this approach, the distinct characteristics of tremulous motion can be effectively captured and differentiated from voluntary motion.\

Furthermore, the remaining features are defined below:

| $Kurtosis k=\frac{1}{N}\sum_{i=1}^{N} \left( \frac{x_{i}-\bar{x}}{\sigma\left( x \right)} \right)^{4}$ |  |
| --- | --- |
| $Peak-Magnitude-to-RMS Ratio=\frac{\left\vert\left\vert x \right\vert\right\vert_{\infty}}{\sqrt{\frac{1}{N}\sum_{i=1}^{N} \left( x_{i} \right)^{2}}}$ |  |
| $Sample entropy= -\log(\frac{\sum A_{i}}{\sum B_{i}})$ |  |
|  |  |

Where $N$ denotes the total number of samples in a given signal $x$, $A_{i}$ and $B_{i}$denotes the number of matches of length m+1 and m respectively.

Kurtosis is measuring the “tailedness” or "peakedness" of the probability distribution. A higher kurtosis indicates that the signal has more than one peak and has a more complex and potentially non-normal distribution than a simple Gaussian distribution. While the Peak-Magnitude-to-RMS ratio is the ratio of the largest absolute value in the input signal to the root-mean-square value of the input signal. The ratio is often used to identify the signal with abnormal or anomalous behavior. Lastly, Sample entropy is a measurement of the regularity of a time series signal, and it is the negative logarithm of probability that two sequences in a time series signal are similar for m and m+1 points where m is the embedded dimension which is chosen as 3 in this project.

## Convolutional LSTM

The convolutional long short-term memory (LSTM) network consists of two components, the first component will be convolutional neural networks (CNN) which is built for features extraction purposes, while the second component is an LSTM network that is used for classification.

CNN can consist of several layers, including convolutional layers, pooling layers, and fully connected layers. These layers work together to extract features from the input data by applying sets of filters to the data. This feature extraction process is based on the recognition of patterns and relationships between the different elements of the input data. For this project, the CNN architecture will comprise a 1D convolutional layer with 30 filters and a kernel size of 20, followed by a max-pooling layer. Then, another 1D convolutional layer with 30 filters and a kernel size of 10, followed by another max-pooling layer and a fully connected layer will be included. The ReLU function will serve as the activation for the convolutional layer, while the stochastic gradient descent algorithm with learning rate of 0.05, momentum of 0.9, and decay of 0.0004 will be utilized as the optimizer. Moreover, the 64 unit of LSTM will be attached to the fully connected layer to carry out the classification task using the features extracted by the CNN. Figure 7 below shows the architecture of CNN.

## Machine Learning models

Support Vector Machines (SVM), a widely acclaimed supervised machine learning technique for classification and regression tasks, was employed in this study. SVM aims to find the optimal hyperplane with the maximum margin that separates the data into distinct classes in the N-dimensional feature space. Various kernel functions, including Radial Basis Function (RBF), polynomial, and sigmoid, can be employed to find the optimal hyperplanes that effectively separate the data into classes. In the present work, an SVM classifier with RBF kernel was utilized to distinguish between voluntary and tremulous motions. The regularization parameter was set to 1, which governs the trade-off between minimizing training error and model complexity. The gamma parameter, which controls the influence of individual samples on the kernel function, was calculated as 1 / (n_features * X.var()), where n_features is the number of features, and X.var() is the variance of the input data. Consequently, the kernel scale is equal to n_features * X.var(). The target variable was encoded as 0 and 1 for voluntary and tremulous motion respectively.

K-Nearest Neighbour (KNN) is a widely used non-parametric supervised machine learning algorithm for classification and regression tasks. In KNN, the input data is represented as points in a multi-dimensional feature space. To classify a sample, a hyperparameter K needs to be set prior to the training process. The classification of a training sample is based on the majority class of the k-nearest training samples closest to that query point. The distance metric is another important hyperparameter that needs to be considered in KNN. To calculate the distance between the input data and other data, commonly used distance metrics are Euclidean distance or Hamming distance. For the KNN developed in this project, Euclidean distance will be used to calculate the distance, and the value of K will be set to 3.

Naïve Bayes is a probabilistic supervised machine learning algorithm that applying Bayes’ Theorem with the assumption of strong independence between the features. The classification task is performed by calculating the conditional probability of each class label based on the input features, and then predicting the class with the highest probability. To train a Naïve Bayes classifier, a training dataset with known class label is required. After calculating the conditional probability of each feature given each class label, the algorithm utilizes these probabilities to compute the posterior probability of each class label based on the input features. In this project, a Gaussian Naïve Bayes will be developed to perform the classification using the features extracted from the IMFs.
